# Anti-Amoebic Properties of Carbonyl Thiourea Derivatives 

**DOI:** 10.3390/molecules19045191

**Published:** 2014-04-22

**Authors:** Maizatul Akma Ibrahim, Mohd Sukeri Mohd Yusof, Nakisah Mat Amin

**Affiliations:** School of Fundamental Science, Universiti Malaysia Terengganu, Kuala Terengganu, Terengganu 21030, Malaysia; E-Mails: maironan@yahoo.com (M.A.I.); mohdsukeri@umt.edu.my (M.S.M.Y.)

**Keywords:** thiourea derivatives, anti-amoebic agent, *Acanthamoeba*, *Acanthamoeba* keratitis, morphology, membrane integrity

## Abstract

Thiourea derivatives display a broad spectrum of applications in chemistry, various industries, medicines and various other fields. Recently, different thiourea derivatives have been synthesized and explored for their anti-microbial properties. In this study, four carbonyl thiourea derivatives were synthesized and characterized, and then further tested for their anti-amoebic properties on two potential pathogenic species of *Acanthamoeba*, namely *A. castellanii* (CCAP 1501/2A) and *A. polyphaga* (CCAP 1501/3A). The results indicate that these newly-synthesized thiourea derivatives are active against both *Acanthamoeba* species. The IC_50_ values obtained were in the range of 2.39–8.77 µg·mL^‑1^ (9.47–30.46 µM) for *A. castellanii* and 3.74–9.30 µg·mL^‑1^ (14.84–31.91 µM) for *A. polyphaga*. Observations on the amoeba morphology indicated that the compounds caused the reduction of the amoeba size, shortening of their acanthopodia structures, and gave no distinct vacuolar and nuclear structures in the amoeba cells. Meanwhile, fluorescence microscopic observation using acridine orange and propidium iodide (AOPI) staining revealed that the synthesized compounds induced compromised-membrane in the amoeba cells*.* The results of this study proved that these new carbonyl thiourea derivatives, especially compounds M1 and M2 provide potent cytotoxic properties toward pathogenic *Acanthamoeba* to suggest that they can be developed as new anti-amoebic agents for the treatment of *Acanthamoeba* keratitis.

## 1. Introduction

*Acanthamoeba* is one of the free-living amoebae that are widely distributed in the environment [1]. This amoeba genus is among the most common protozoa to be found in soil and water samples [2]. *Acanthamoeba* is known as the causative agent for a sight-threatening disease, *Acanthamoeba* keratitis. This eye infection is recognized as one of the most challenging and severe ocular parasitic diseases [3]. The *Acanthamoeba* species which have been reported to cause *Acanthamoeba* keratitis are *A. castellanii*, *A. polyphaga*, *A. hatchetti*, *A. culbertsoni*, *A. rhysodes*, *A. griffini*, *A. quina*, and *A. lugdunensis* [4]. An effective medical therapy for treating the infection is currently not available. Several antiseptics such as chlorhexidine gluconate and polyhexamethylene biguanide have been used to lessen the symptoms [5,6], but they are not specifically designed to treat the ocular disease, thus side effects are frequently reported [7,8]. Some surveys showed that *Acanthamoeba* are resistant to these agents, which make them less effective [9,10] especially at later stages of infection. Therefore, new potential agents are in high demand to assist the current treatment of *Acanthamoeba* keratitis.

Since synthetic organic compounds are being widely designed nowadays in parallel with the development of combinatorial chemistry and compound libraries, they could be exploited for the development of new drugs. Some synthetic compounds such as quinoxaline derivatives and thiosemicarbazone analogs were investigated on the cells of *Entamoeba histolytica* and found to display beneficial properties which can be developed as anti-amoebic agents [11,12]. Thiourea, which is one of the earliest synthetic organic compounds, has been globally used directly and indirectly due to its ready availability. This factor has attracted researchers to evaluate thiourea-based compounds from their safety point of view [13] and potential medical properties [14,15,16].

Previous studies have shown the potential of certain thiourea derivatives as anti-microbial agents [17,18]. Drugs which are based on thiourea have also been used clinically to treat patients of tuberculosis [19] and thyroid conditions [20]. Therefore, in the present study, four new carbonyl thiourea derivatives were synthesized and characterized, and could possibly be developed as new agent to treat *Acanthamoeba* keratitis after their anti-amoebic properties were examined. Cytotoxicity tests which involved investigation of the inhibition of amoeba population and disruption of the amoeba membrane integrity caused by the compounds were conducted. Microscopic observation was also carried out to examine the morphological alterations in the amoeba cells caused by these newly-synthesized compounds.

## 2. Results and Discussion

### 2.1. Preparation of Carbonyl Thiourea Derivatives

**Scheme 1:**
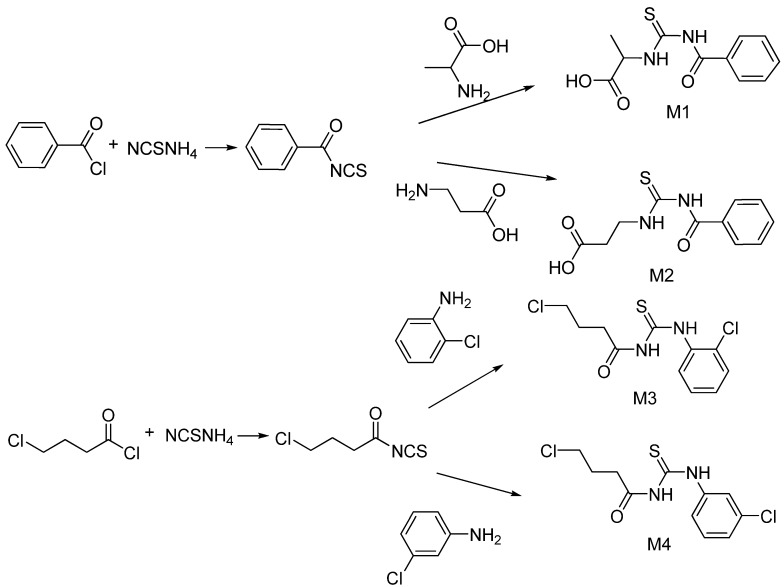
Synthesis of carbonyl thiourea compounds.

The preparation of compounds **M1**–**M4** is shown in Scheme 1 [21,22], while the compounds obtained and their molecular weights are listed in Table 1.

### 2.2. Anti-Amoebic Properties: IC_50_ Values

Experiments were carried out to analyze the *in vitro* anti-amoebic activity of the four newly-synthesized carbonyl thiourea derivatives on two pathogenic species of *Acanthamoeba*, namely *A. castellanii* (CCAP 1501/2A) and *A. polyphaga* (CCAP 1501/3A). The amoebae were obtained from the UK Culture Collection of Algae and Protozoa (CCAP, Argyll, UK). The IC_50_ values which were obtained from the absorbance readings and represented in non-linear sigmoidal dose-response curve derived from GraphPrism software are presented in Table 2.

**Table 1:** The molecular structures of the newly-synthesized carbonyl thiourea derivatives.

Code	Chemical name	MW	Molecular structure
**M1**	2-(3-Benzoylthioureido)propanoic acid	252.29	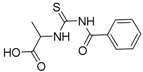
**M2**	3-(3-Benzoylthioureido)propanoic acid	252.29	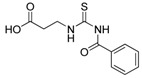
**M3**	*N*-(2-Chlorophenyl)-*N'-*(4-chlorobutanoyl)thiourea	291.20	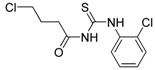
**M4**	*N*-(3-Chlorophenyl)-*N'*-(4-chlorobutanoyl)thiourea	291.20	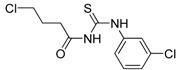

**Table 2:** The IC_50_ values of the newly-synthesized thiourea derivatives against *Acanthamoeba* and their comparative strength (%) as compared with the positive control, chlorhexidine.

Compound	IC_50_ (µg·mL^−1^)				
	*A. castellanii* (CCAP 1501/2A)			*A. polyphaga* (CCAP 1501/3A)	
**M1**	2.39 ± 0.24			3.74 ± 0.44	
**M2**	3.34 ± 0.41			3.76 ± 0.27	
**M3**	8.07 ± 0.65			8.52 ± 0.81	
**M4**	8.87 ± 0.27			9.30 ± 0.55	
	**IC_50_ (µM)**				
	***A. castellanii*** **(CCAP 1501/2A)**	**Percentage of strength (%)**	***A. polyphaga*** **(CCAP 1501/3A)**		**Percentage of strength (%)**
**M1**	9.47	73.5	14.84		52.4
**M2**	13.24	52.6	14.90		52.1
**M3**	27.70	25.1	29.25		26.6
**M4**	30.46	22.9	31.91		24.3
**Chlorhexidine**	6.96	100.0	7.77		100.0

All compounds used in the present study have high anti-amoebic activity against *Acanthamoeba* with IC_50_ values in the range from 2.39 to 8.87 µg·mL^−1^ for *A. castellanii*, and 3.74 to 9.30 µg·mL^−1^ for *A. polyphaga*, which are equivalent to 9.47–30.46 μM and 14.84–31.91 μM respectively (Table 2). These derivatives were thus observed to be active against *A. castellanii* and moderately active toward *A. polyphaga* based on compounds classification for the protozoan cells proposed by Deharo [23]. This means that *A. castellanii* is more susceptible towards the series of newly-synthesized carbonyl thiourea compounds compared to *A. polyphaga*. McBride *et al.* [24], in their study of drug efficacy, also noted that *A. polyphaga* was more resistant compared to *A*. *castellanii*, confirming the data obtained in the present study. The strength of chlorhexidine, a positive control in this study against *Acanthamoeba* was considered as 100% and its IC_50_ value was 6.96 μM for *A. castellanii* and 7.77 μM for *A. polyphaga.* The *t*-test analysis for the absorbance readings of untreated and treated amoebae showed statistically significant differences (*p* < 0.05).

Thiourea in its basic structure has one sulfur atom, which has six valence electrons and its electronic configuration is similar to that of oxygen [25]. The amino acid type of thiourea derivatives labeled as **M1** and **M2** in this study showed higher anti-amoebic activity. Their strength as compared with chlorhexidine against both species of *Acanthamoeba* is shown in Table 2. This indicates that the amino acid moieties in **M1** and **M2** could enhance the activity of thiourea derivatives against *Acanthamoeba* cells. Fustero *et al.* [26] supported this finding by highlighting that in general, amino acid derivatives of compounds can exhibit a variety of biological properties. Meanwhile, Ye *et al.* [27] emphasized that amino acids derivatives in compounds would give them a hydrophilic moiety which leads to high selectivity toward receptors. This suggests that the mechanism of action for the proposed thiourea derivatives toward the protozoan parasite *Acanthamoeba* should focus on the hydrophobicity of thiourea molecules to explain their actions. The suggested drug-receptors for the compounds’ main target in the amoeba cells are the transport proteins that are distributed throughout the cell membrane. This explains that the thiourea chemical molecules’ preliminary penetration into *Acanthamoeba* is through its membrane. However, the detail of the mechanism of action of the amino acid group toward the amoeba cells is poorly understood.

Compounds **M3** and **M4** contain one chloride halogen atom in their benzene rings. The presence of these halogens contributes to the compounds’ activity against *Acanthamoeba*. Patel and Shaikh [28] reported that several compounds containing chlorine atom had better anti-microbial activity compared to compounds without the halogen atom. Furthermore, the presence of chlorine in chlorhexidine was proven to contribute in its anti-amoebic activity. However, the anti-amoebic activity of compounds **M3** and **M4** in this study were non-comparable to **M1** and **M2** that contain amino acid groups which gave stronger in actions against the tested amoeba cells. 

### 2.3. Morphological Changes in Acanthamoeba

**Figure 1:**
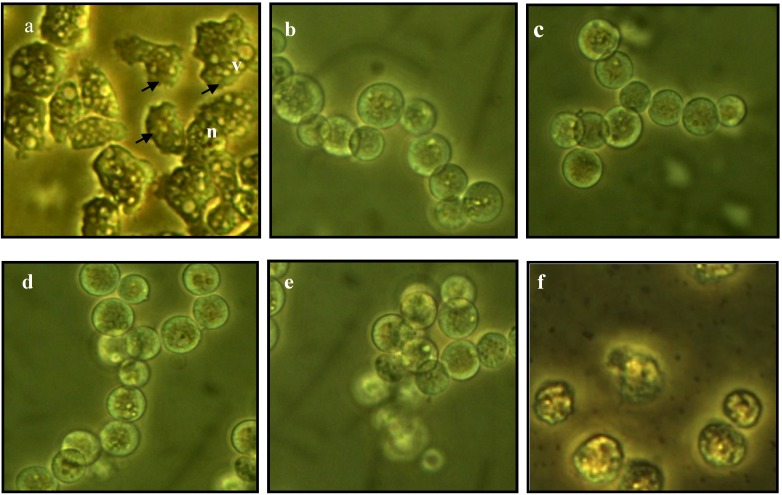
Light microscopy of *A. castellanii* (**a**) Untreated cells with obvious acanthapodia structures on the cells surface (arrows); (**b**) **M1-**treated cells; (**c**) **M2-**treated cells; (**d**) **M3-**treated cells; (**e**) **M4-**treated cells; (**f**) Chlorhexidine-treated cells. Nucleus (**n**); vacuoles (**v**). Magnification 300×.

**Figure 2:**
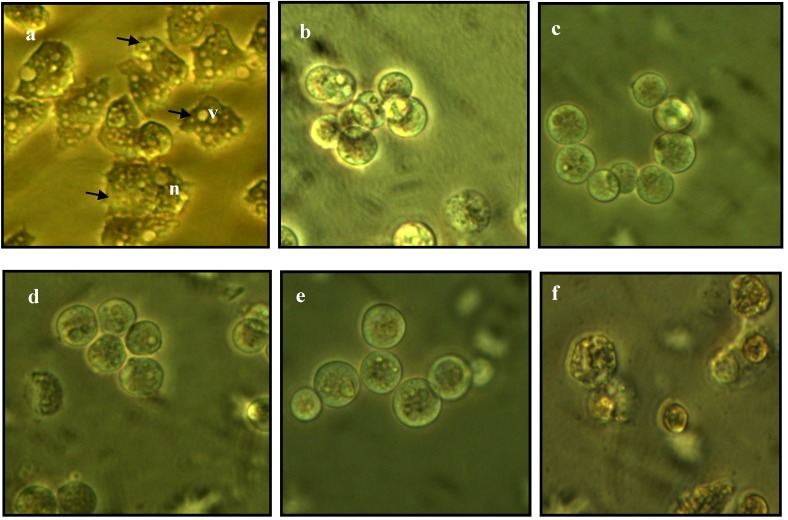
Light microscopy of *A. polyphaga*. (**a**) Untreated cells; (**b**) **M1**-treated cells with acanthapodia structures on the cells surface (arrows); (**c**) **M2**-treated cells; (**d**) **M3**-treated cells; (**e**) **M4**-treated cells; (**f**) Chlorhexidine-treated cells. Nucleus (**n**); vacuoles (**v**). Magnification 300×.

The morphological structures of untreated, as well as thiourea- and chlorhexidine-treated *Acanthamoeba* of both species are shown in Figure 1 and Figure 2. The untreated cells exhibited distinct structures of acanthopodia, vacuoles and nuclei. Meanwhile, for the thiourea-treated *Acanthamoeba*, vacuoles and nucleus were not apparent, and the cells were also observed to be smaller in size. The morphology of treated *Acanthamoeba* became rounded due to shortening and loss of their acanthopodia structures, which eventually caused the amoeba cells to detach from the well’s surface and float in the culture medium.

Acanthopodia are important for amoebas’ adherence to surfaces, cellular movements and capturing food particles [29]. The alteration of acanthopodia structures as induced by thiourea derivatives in the present study indicates a significant effect on the biology of protozoan cells. These structures also play a key role in *Acanthamoeba* pathogenesis of amoebic keratitis by modulating a binding to the corneal epithelium of the human host. This leads to secondary events such as interference with host intracellular signaling pathways and toxic secretions from *Acanthamoeba* which phagocytose host cells that ultimately leads to cell death [30]. With impaired acanthopodia, the pathogenesis of *Acanthamoeba* will be affected. The thiourea-treated cells were also observed without distinct nucleus. Prominent vacuoles were seen in healthy *Acanthamoeba* cells but not in the treated amoeba, where its function is to expel water as well as be involved in osmotic regulation that helps the cells move and capture food [31].

After treatment with the thiourea derivatives *Acanthamoeba* were also reduced in size and became rounded and displayed a cystic appearance. This suggests that the compounds induce encystment in *Acanthamoeba*. Encystment is a process that involves a drastic reorganization of the subcellular structure of the amoeba cell in which acanthopodia, nucleus and vacuoles disappear. In this stage, the trophozoite condensed itself into a rounded structure with a decrease in cytoplasmic mass, whereby excess food, water and particulate matter are expelled. This was accompanied by the synthesis of a structurally complex double layer wall cyst to help amoeba survive in hostile conditions [32]. Throughout the course of the encystment process, the respiration rates and intracellular ATP levels of cells will be diminished. The cellular levels of RNA, proteins, triacylglyceridases and glycogen will also decline substantially. This would result in a decreased cellular volume and dry weight [33]. As a conclusion, with the treatment of the carbonyl thiourea, *Acanthamoeba* became inactivated, making them unable to affect the host cells during pathogenesis. Chlorhexidine gave comparable effects on the morphology of *Acanthamoeba* as shown by the thiourea derivatives.

### 2.4. Integrity of Acanthamoeba Membrane

**Figure 3:**
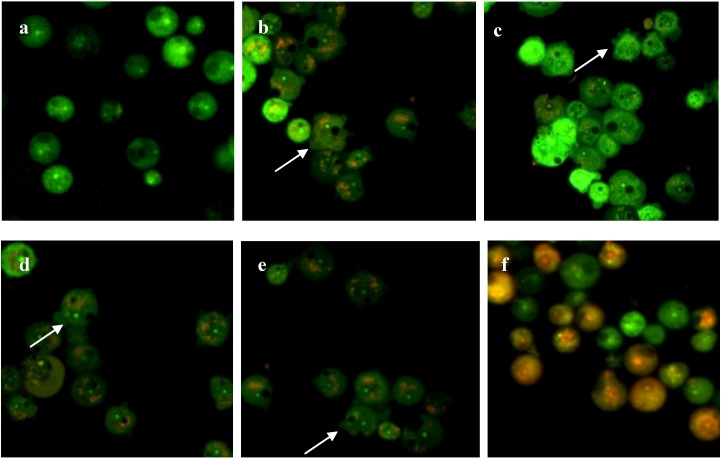
Fluorescence micrographs of *A. castellanii* stained with AO/PI. (**a**) Untreated cells; (**b**) **M1**-treated cells; (**c**) **M2**-treated cells; (**d**) **M3**-treated cells; (**e**) **M4**-treated cells; (**f**) Chlorhexidine-treated cells. Membrane blebbings were observed in all compound-treated amoebae (arrows). Magnification 300×.

**Figure 4:**
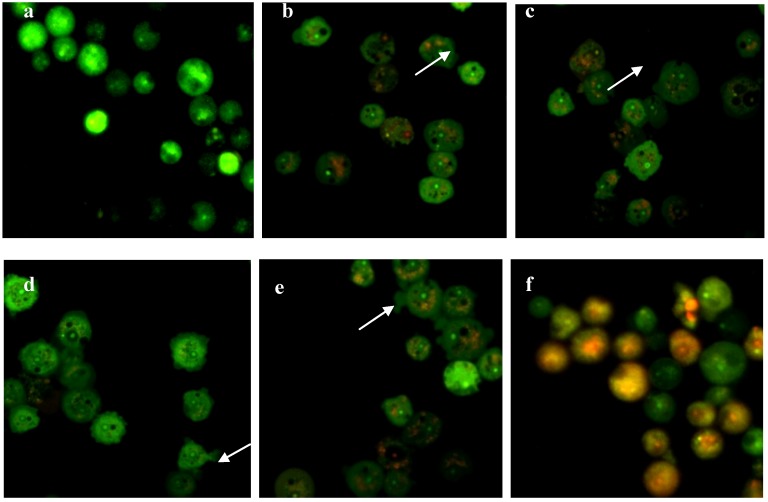
Fluorescence micrographs of *A. polyphaga* stained with AO/PI. (**a**) Untreated cells; (**b**) **M1**-treated cells; (**c**) **M2**-treated cells; (**d**) **M3**-treated cells; (**e**) **M4**-treated cells; (**f**) Chlorhexidine-treated cells. Membrane blebbings were observed in all compound-treated amoebae (arrows). Magnification 300×.

*Acanthamoeba* trophozoites consist of a plasma membrane which is a thin layer that surrounds the cells and is comprised of phospholipids (25%), proteins (33%), sterols (13%), and lipophosphonoglycans (29%) [31], while the cytoplasm of *Acanthamoeba* possesses large numbers of fibrils, glycogen, lipid droplets, and a variety of lysosomal enzymes such as *α*- and *β*-glycosidases, amylase, *β*-galactosidase, *β*-*N*-acetylglucosaminidase, *β*-glucuronidase, protease, phosphatase, hydrolase acid, RNAse, and DNAse [33]. In all living cells, membrane integrity is essential in maintaining their internal part in order to keep them viable. Compounds with cytotoxic effects would often lead to compromised membrane integrity [34]. Disturbed membrane integrity would disrupt the physiology of the cells’ inner state as well as organelles normal functions. In this study, fluorescence microscopic observation based on a dual staining technique was conducted to evaluate the integrity of the amoeba membrane with the given treatment. Acridine orange/propidium iodide (AO/PI) simultaneous staining was applied to distinguish between cells of intact membrane with compromised-membrane integrity as shown in Figure 3 and Figure 4.

AO is technically an intercalating agent which can bind to the double strand structure of DNA by intercalating inside the double helix structure. It stains cells with green fluorescence under fluorescence microscopy. AO uptake is the result of an active proton pump in the lysosome of healthy cells. High proton concentration gives AO the ability to enter the uncharged lysosome. The stain becomes protonated and later trapped in the organelles of viable cells [35]. AO is defined as a membrane-permeable dye which can readily enter internal parts of *Acanthamoeba* through non-compromised membrane integrity. On the other hand, PI is a cationic and an impermeable dye thus excluded from entering normal healthy cells. PI can only traverse and stain cells’ intracellular components from leakage and pores formed in membranes [36]. According to Arnkt-Jovin and Jovin [37], when PI is bound to nucleic acids, its orange fluorescence is enhanced 20 to 30-fold and can be observed well under a fluorescence microscope.

From these principles, the integrity of *Acanthamoeba* membranes after being treated with thiourea derivatives could be evaluated (Figure 3 and Figure 4). Under fluorescence microscopy, the untreated *Acanthamoeba* appeared as green fluorescent cells, indicating that they were viable cells with intact membrane structures which only allowed the diffusion of AO through their membranes. On the other hand, the thiourea-treated amoebae exhibited membrane blebbing with orange fluorescence bits in their cytoplasms which were distinguishable from the untreated viable cells. Therefore, the four synthetic compounds used in the present study were proven to disrupt the integrity of amoeba membranes. Meanwhile, chlorhexidine-treated *Acanthamoeba* also showed compromised membranes by displaying an orange fluorescence color. However, complete orange fluorescence was observed in cells treated with chlorhexidine, suggesting that the agent caused total leakage of *Acanthamoeba* membranes. Under fluorescence microscopy, when both dyes are used simultaneously on compromised cell membranes, an orange color fluorescence will be emitted from the cells due to stronger action of PI compared to AO [38].

Perrine *et al.* [39] studied the lethal effects of amidine compounds toward *Acanthamoeba* and showed that protonated substituents attached to compounds interact with the amphipathic lipids of amoeba’s plasma membrane bilayer. This could induce the membrane’s structural changes which lead to the modifications of the cell membrane permeability. From this study, it is suggested that the penetration across the *Acanthamoeba* membrane by the compounds reflects the lipophilic properties of the newly-synthesized thiourea derivative compounds. Nakisah *et al.* [40] used the same AO/PI staining technique to explain the mode of cell death promoted by crude extracts from Malaysian marine sponges on *A. castellanii*.

## 3. Experimental

### 3.1. General Information

All the compounds utilized in this work were commercially available Merck, Darmstadt, Germany and use as supplied with no further purification. The infrared spectrum (IR) of the product (KBr pellets) was recorded using a Perkin Elmer Spectrum GX spectrophotometer (Perkin Elmer, Waltham, MA, USA) in the range of 400–4000 cm^−1^. NMR spectra were recorded on a Bruker Ultrashield 400 MHz NMR spectrometer using CDCl_3_ as the solvent.

### 3.2. Synthesis of Carbonyl Thiourea Derivatives

The method to prepare **M1**–**M2** was based on Yusof and Yamin [21], while compounds **M3** and **M4** followed the method of Yusof *et al.* [22] according to the routes shown at Scheme 1. Generally, the carbonyl chloride reacted with ammonium isothiocyanate in acetone resulting carbonylisothiocyanate. The carbonylisothiocyanate then will be reacted with amine derivate and the mixture was put at reflux for 2.5 h then filtered off and left to evaporate at room temperature. For compound **M1** (benzoyl chloride, 2.03 g (14.44 mmol), α-alanin, 1.29 g (14.44 mmol), ammonium thiocyanate, 1.10 g (14.44 mmol); compound **M2**, (benzoyl chloride, 1.9 5 g (13.87 mmol), β-alanin, 1.24 g (13.87 mmol), ammonium thiocyanate, 1.06 g (13.87 mmol); compound **M3**, (4-chlorobutyryl chloride, 2.12 g (15.04 mmol), 2-chloroaniline, 1.92 g (15.04 mmol), ammonium thiocyanate, 1.14 g (15.04 mmol); compound **M4**, (4-chlorobutanoyl chloride, 2.05 g (14.54 mmol), 3-chloroaniline, 1.85 g (14.54 mmol), ammonium thiocyanate, 1.11 g (14.54 mmol).

### 3.3. Characterization of the Newly-Synthesized Carbonyl Thiourea Derivatives

*2-(3-Benzoylthioureido)propanoic acid* (**M1**). The title compound was obtained as colourless crystals in 38% yield after recrystallization from ethanol; IR (KBr pellets, υ/cm^−1^): 3389.22 (O-H), 3234.82 (N-H), 1772.31 (C=O), 1355.82 (C-N), 782.93 (C=S); 1H-NMR (400.130 MHz, DMSO-*d*_6_, ppm): 1.42 (3H, d, CH_3_), 3.52 (1H, dd, CH), 7.27 (1H, dd, C_6_H_4_), 7.65 (2H, m, C_6_H_4_), 7.88 (2H, d, C_6_H_4_), 11.44 (1H, s, NH), 12.01 (1H, s, OH), 12.20 (1H, s, NH); ^13^C-NMR (100.613 MHz, DMSO-*d*_6_; ppm): 17.23 (CH_3_), 62.32 (NHCH), 126.82 (CH_Ar_), 129.09 (CH_Ar_), 130.24 (NHC_Ar_), 172.02 (C=O), 175.52 (C=O_OH_), 180.43 (C=S).

*3-(3-Benzoylthioureido)propanoic acid* (**M2**). The title compound was obtained as colourless crystals in 52% yield after recrystallization from ethanol; IR (KBr pellets, υ/cm^−1^): 3324.61 (O-H), 3203.79 (N-H), 1794.05 (C=O), 1365.13 (C-N), 774.02 (C=S); 1H-NMR (400.130 MHz, DMSO-*d*_6_, ppm): 2.63 (2H, dd, NHCH_2_CH_2_), 3.67 (2H, dd, NHCH_2_CH_2_), 7.29 (1H, dd, C_6_H_4_), 7.64 (2H, m, C_6_H_4_), 7.87 (2H, d, C_6_H_4_), 11.54 (1H, s, NH), 12.03 (1H, s, OH), 12.23 (1H, s, NH); ^13^C-NMR (100.613 MHz, DMSO-*d*_6_, ppm): 34.25 (NHCH_2_CH_2_), 43.18 (NHCH_2_), 127.64 (CH_Ar_), 130.29 (CH_Ar_), 133.71 (NHC_Ar_), 172.84 (C=O), 175.61 (C=O_OH_), 181.32 (C=S).

*N-(2-Chlorophenyl)-N'-(4-chlorobutanoyl)thiourea* (**M3**). The title compound was obtained as colorless crystal in 73% yield after recrystallization from dimethylformamide; IR (KBr pellets, υ/cm^−1^): 3164.31 (N-H), 1697.18(C=O), 1337.40(C-N), 723.53 (C=S); 1H-NMR (400.130 MHz, DMSO-*d*_6_, ppm): 2.02 (2H, m, COCH_2_CH_2_CH_2_Cl), 2.65 (2H, t, COCH_2_CH_2_CH_2_Cl), 3.66 (2H, t, COCH_2_CH_2_CH_2_Cl), 7.25 (1H, d, C_6_H_4_), 7.56 (1H, t, C_6_H_4_), 7.59 (1H, t, C_6_H_4_), 8.01 (1H, d, C_6_H_4_), 11.51 (1H, s, NH), 12.45 (1H, s, NH); ^13^C-NMR (100.613 MHz, DMSO-*d*_6_, ppm): 27.28 (COCH_2_CH_2_CH_2_Cl), 33.53 (COCH_2_CH_2_CH_2_Cl), 45.01 (COCH_2_CH_2_CH_2_Cl), 115.94 (CH_Ar_), 116.10 (CH_Ar_), 127.41 (NHC_Ar_), 134.69 (ClC_Ar_), 175.92 (C=O), 180.12 (C=S).

*N-(3-Chlorophenyl)-N'-(4-chlorobutanoyl)thiourea*, **M4**. The title compound was obtained as colourless crystal in 75% yield after recrystallization from dimethylformamide; IR (KBr pellets, υ/cm^−1^): 3165.88 (N-H), 1694.05 (C=O), 1325.09 (C-N), 780.65 (C=S); 1H-NMR (400.130 MHz, DMSO-*d*_6_, ppm): 2.03 (2H, m, COCH_2_CH_2_CH_2_Cl), 2.64 (2H, t, COCH_2_CH_2_CH_2_Cl), 3.69 (2H, t, COCH_2_CH_2_CH_2_Cl), 7.24 (1H, d, C_6_H_4_), 7.29 (1H, t, C_6_H_4_), 7.62 (1H, d, C_6_H_4_), 7.96 (1H, s, C_6_H_4_), 11.47 (1H, s, NH), 12.42 (1H, s, NH). ^13^C-NMR (100.613 MHz, DMSO-*d*_6_, ppm): 27.25 (COCH_2_CH_2_CH_2_Cl), 45.04 (COCH_2_CH_2_CH_2_Cl), 33.54 (COCH_2_CH_2_CH_2_Cl), 115.70 (CH_Ar_), 115.92 (CH_Ar_), 127.31 (NHC_Ar_), 134.67 (ClC_Ar_), 175.81 (C=O), 179.89 (C=S).

### 3.4. Determination of IC_50_ Values

Thiourea derivatives were prepared by dissolving 1 mg of compound in 10 µL absolute DMSO (Fisher Scientific, Schwerte, UK) and added with 990 µL sterile culture media, to make a 1 mg·mL^−1^ solution. Dissolution was facilitated by mild sonication in a sonicator bath (Branson, CT, USA) for two minutes. Then, 100 µL of the 1 mg·mL^−1^ samples were further diluted with 900 µL of culture media to produce compound stocks of 100 µg·mL^−1^ with 0.1% DMSO. These thiourea compounds solutions were freshly prepared before conducting every experiment. The experiment was conducted in 96-well plates (Nunc, Schwerte, Germany). Nine different concentrations of compounds were prepared to give final concentrations of compounds as follows: 100, 50, 25, 12.5, 6.25, 3.13, 1.56, 0.78 and 0.39 µg·mL^−1^. Each concentration was prepared in three replicates. Chlorhexidine gluconate (Raza Manufacturing, Kuala Lumpur, Malaysia) which is a common agent used for treatment of amoebic keratitis infections was used as the positive control. The nine final concentrations of chlorhexidine used for the assays were as follows: 200, 100, 50, 25, 12.5, 6.25, 3.13, 1.56 and 0.78 µM.

The number of viable *Acanthamoeba* for treatment was calculated by using a hemocytometer with trypan blue. A calculated amount of ~10^4^ viable cells·mL^−1^ was used as the number or concentration of *Acanthamoeba* of which the cells would reach their confluence stage after 72 h of incubation without excessive growth [41]. Negative control was 10^4^ cells·mL^−1^ of healthy *Acanthamoeba* without any treatment. The plates were later incubated at 30 °C for 72 h. After incubation, the staining process was done following Wright’s technique [42]. The final solutions from all wells were read for their absorbance at 490 nm by ELISA microplate reader (Tecan, Victoria, Australia). The readings were plotted in GraphPad Prism software version 5.03 (GraphPad Inc., San Diego, CA, USA) to give a non-linear sigmoidal dose-response curve. The cytotoxicity was expressed as the IC_50_ value that represents the concentration of a compound that is required for inhibition of 50% of an *Acanthamoeba* population *in vitro*. A *t*-test (SPSS, version 11.5., SSPS Inc., Armonk, NY, USA) was done to compare the mean values between untreated and treated cultures with *p* < 0.05 considered as statistically significant.

### 3.5. Observation of Changes in Acanthamoeba Morphology

*Acanthamoeba* both untreated and treated with the compounds were observed for their morphological changes. *Acanthamoeba* (10^4^ cells·mL^−1^) were treated with the thiourea compounds and the positive control (chlorhexidine) at their IC_50_ concentration in 6-well-plates, which were then incubated at 30 °C for 72 h. After the incubation, the morphology of *Acanthamoeba* was observed directly from the well plates under an inverted microscope (Leica Leitz, Wetzlar, Germany). Images were captured by using Image Master Video Test Package (Trioptics, Wetzlar, Germany) software.

### 3.6. Evaluation of Acanthamoeba Membrane Integrity

*Acanthamoeba* were adjusted to 10^4^ cells in 1 mL culture media prior to the treatment with thiourea compounds and chlorhexidine, at their IC_50_ concentration in 25-cm^2^ tissue culture flasks and later incubated at 30 °C for 72 h. After the incubation, the cell suspension was resuspended, harvested and transferred into Eppendorf tubes for AO/PI staining. Stock solution for AO/PI staining was prepared by adding AO (2 µL, 1 mg·mL^−1^, Sigma, St. Louis, MO, USA) and PI (2 µL, 1 mg·mL^−1^, Sigma) to give a mixture of 1:1 (v/v) ratio in 996 µL phosphate buffered saline (PBS, Sigma). The AO/PI staining protocol followed the technique by Mascotti *et al.* [43]. Both dyes are light sensitive therefore they were handled in a dark room. The harvested *Acanthamoeba* cells were centrifuged at 1,000 rpm for 5 min at 4 °C. The supernatant were discarded and pellets were washed with PBS and re-centrifuged at 1,000 rpm for 5 min. The fresh pellets were mixed with 20 µL of AO/PI staining from the stock and transferred onto microscope slides and viewed under a fluorescence microscope (Leica Dmire, Wetzlar, Germany) in dark condition. Images were captured by Image Master Video Test Package software (Trioptics).

## 4. Conclusions

The results of this study indicate that the newly-synthesized carbonyl thiourea derivatives provide promising anti-*Acanthamoeba* properties against pathogenic *A. castellanii* and *A. polyphaga*. Based on their low IC_50_ values the compounds 2-(3-benzoylthioureido)propanoic acid (**M1**) and 3-(3-benzoylthioureido)propanoic acid (**M2**) exhibited stronger anti-amoebic activity compared to the other tested compounds used, and this finding correlates with the presence of amino acids groups in their molecular structures. All thiourea derivatives used in this study were proven to cause *Acanthamoeba* to become inactive, and can disrupt the integrity of the amoeba cell membrane. Therefore, these new carbonyl thiourea derivatives can be suggested as future anti-amoebic agents.
